# *Announcement*: Final 2016–17 Influenza Vaccination Coverage Estimates Available Online

**DOI:** 10.15585/mmwr.mm6638a5

**Published:** 2017-09-29

Final 2016–17 influenza season vaccination coverage estimates for selected local areas, states, U.S. Department of Health and Human Services regions, and the United States overall are available online at FluVaxView (https://www.cdc.gov/flu/fluvaxview/). The online information includes estimates of the cumulative percentage of persons receiving influenza vaccination through the end of each month during July 2016–May 2017.

Analyses were conducted using National Immunization Survey–Flu influenza vaccination data for children aged 6 months–17 years and Behavioral Risk Factor Surveillance System influenza vaccination data for adults aged ≥18 years. Estimates are provided by age group and race/ethnicity. These estimates are presented in interactive reports (https://www.cdc.gov/flu/fluvaxview/interactive.htm) and are complemented by an online summary report (https://www.cdc.gov/flu/fluvaxview/coverage-1617estimates.htm).

